# Preparation of immobilized arylsulfatase on magnetic Fe_3_O_4_ nanoparticles and its application for agar quality improvement

**DOI:** 10.1002/fsn3.2446

**Published:** 2021-07-07

**Authors:** Chenghao Zhang, Zedong Jiang, Hebin Li, Hui Ni, Mingjing Zheng, Qingbiao Li, Yanbing Zhu

**Affiliations:** ^1^ College of Food and Biological Engineering Jimei University Xiamen China; ^2^ Fujian Provincial Key Laboratory of Food Microbiology and Enzyme Engineering Xiamen China; ^3^ Research Center of Food Biotechnology of Xiamen City Xiamen China; ^4^ Key Laboratory of Systemic Utilization and In‐depth Processing of Economic Seaweed Xiamen Southern Ocean Technology Center of China Xiamen China; ^5^ Xiamen Medical College Xiamen China

**Keywords:** agar desulfation, arylsulfatase, immobilized enzyme, magnetic nanoparticle, tannic acid

## Abstract

The presence of sulfate groups in agar compromises the agar quality by affecting the crosslinking during gelling process. Some arylsulfatases can catalyze the hydrolysis of sulfate bonds in agar to improve the agar quality. Immobilized arylsulfatases prove beneficial advantages for their industrial applications. Here, a previously characterized mutant arylsulfatase K253H/H260L was immobilized on the synthesized magnetic Fe_3_O_4_ nanoparticles after functionalization by tannic acid (MNPs@TA). The surface properties and molecular structures of the immobilized arylsulfatase (MNPs@TA@ARS) were examined by scanning electron microscopy and Fourier transform infrared spectroscopy. Enzymatic characterization showed that MNPs@TA@ARS exhibited shifted optimal temperature and pH with deviated apparent *K_m_* and *V_max_* compared to its free counterpart. The immobilized arylsulfatase demonstrated improved thermal and pH stability and enhanced storage stability with modest reusability. In addition, MNPs@TA@ARS displayed enhanced tolerance to various inhibitors and detergents. The utilization of the immobilized arylsulfatase for agar desulfation brought the treated agar with improved quality.

## Graphical abstract

An arylsulfatase of marine bacterial origin was immobilized on the magnetic nanobeads. The immobilized arylsulfatase had enhanced thermal and pH stability. After treatment of agar by the immobilized enzyme, agar had equivalent quality to agarose.

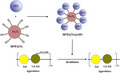

## 1 INTRODUCTION

Agar is the major cell wall component of red algae such as genera of *Gracilariopsis* and *Gelidium*, which provide the abundant raw materials for the development of agar products (Abraham et al., 2018). It has been widely used in biotechnology, food and pharmaceutical industries. Agar consists of agarose and agaropectin, and agarose accounts for more than 70% (Torres et al., 2019). Agarose is a polysaccharide composed of agarobiose, a disaccharide made from D‐galactose and 3,6‐anhydro‐L‐galactopyranose (3,6‐AG). On the other hand, the minority agaropectin is slightly different from agarose, with modifications on the hydroxyl groups of 3,6‐AG, mostly by side groups such as sulfoxy, methoxy, and pyruvate residues (Fu & Kim, 2010). Meaningfully, the presence of sulfate groups in agaropectin compromises the agar quality as they affect the gel strength by interfering with the crosslinking during the gelation process (Zhang et al., 2019).

Arylsulfatase (arylsulfate sulfohydrolase; EC3.1.6.1), existing in diverse organisms ranging from bacteria to mammals, is a type of sulfatase enzyme that can function to cleave the arylsulfate ester bond between aryl group and inorganic sulfate (Gardner & Senwo, 2019). A few studies have uncovered arylsulfatases from different bacterial strains, functioning specifically against sulfate ester bonds in agar and consequently enhancing the gelling strength of agar (Stressler et al., 2016; Wang et al., 2016). Alkaline treatment is the conventional method for the sulfate group removal from agar (Zhang et al., 2019). To meet the growing demand for high‐quality agar in food and bioengineering industries, it is urgent to develop highly efficient and eco‐friendly approach (Torres et al., 2019). The application of arylsulfatase treatment greatly facilitates the structure modification in an environmentally friendly manner as well as in a more efficient and specific way (Cregut & Rondags, 2013). A handful of foreseeable factors constraining the industrial application of arylsulfatases include their shortage of stability, reusability, and convenient separation from the reaction (Mohamad et al., 2015). The immobilized enzymes have proven to show enzymatic and operational advantages over free enzymes in industrial and medical applications (Basso & Serban, 2019). Classical and newly developed materials have been exploited to immobilize enzymes through physical adsorption, entrapment, covalent bonding, and crosslinking, with the enzyme and supporting matrix of choice to serve different applications (Sirisha et al., 2016). Magnetic iron oxide nanoparticles are attractive vehicles to load enzyme normally after surface functionalization (Atacan et al., 2017; Jiang et al., 2021). They have added beneficial values to the immobilized enzyme, especially owing to their large ratio of surface area to volume, the presence of functional groups, and the simple separation (Bilal et al., 2019; Zdarta et al., 2018). Few studies have been conducted on immobilization of arylsulfatase onto solid supporting carriers including activated agarose gel (Toennes & Maurer, 1999) and carboxyl functionalized magnetic nanoparticles (Xiao et al., 2017), conferring the stability, reusability, and functional efficiency of the conjugated enzymes.

In our previous study, a mutant arylsulfatase with two point mutations of K253H and H260L has been characterized and it shows enhanced thermal stability (Zhu et al., 2018). In this study, tannic acid‐functionalized magnetic Fe_3_O_4_ nanoparticles were prepared and used as the carrier for immobilization of the mutant arylsulfatase of K253H/H260L. The morphology and structure of the immobilized arylsulfatase were characterized by scanning electron microscopy (*SEM*), transmission electron microscopy (TEM), and Fourier transform infrared spectroscopy (FTIR). Furthermore, the catalytic properties of the immobilized arylsulfatase were investigated, and the stability and reusability were evaluated accordingly. In addition, the immobilized arylsulfatase was practically monitored for its desulfation activity against agar by the physicochemical and morphological characterization of the treated agar. Our study explores the possibility of the immobilized arylsulfatase for agar desulfation to improve agar quality.

## 2 MATERIALS AND METHODS

### 2.1 Bacteria and reagents

The transformed *E. coli* BL21 (DE3) cells harboring the expression construct of a mutant arylsulfatase (K253H/H260L; recombination with *Bam*HI and *Hin*dIII sites in pET‐28α vector) was obtained from our laboratory stocks (Zhu et al., 2018). *p*‐nitrophenyl sulfate (*p*NPS) was purchased from Sigma‐Aldrich (Saint Louis, MO, USA). Other chemicals of analytical grade were ordered from Sinopharm Chemical Reagent Co., Ltd. (Shanghai, China).

### 2.2 Arylsulfatase protein expression and purification

The *E. coli* cells for expression of arylsulfatase were refreshed and cultured, and the His‐tagged recombinant protein was purified by affinity chromatography using Ni sepharose 6 Fast Flow (GE Healthcare Life Sciences) with reference to the procedures described (Zhu et al., 2018). The purified protein was eluted from the resin and recovered in 50 mM Tris‐HCl (pH 7.5) by equilibrium dialysis.

### 2.3 Immobilization of arylsulfatase on magnetic Fe_3_O_4_ nanoparticles

Magnetic Fe_3_O_4_ nanoparticles (MNPs) were prepared following the method described to co‐precipitate Fe^2+^ and Fe^3+^ under alkaline condition (Atacan & Ozacar, 2015). The MNPs were further functionalized with 2% (w/v) of tannic acid (TA) at 5℃ for 2 hr to obtain the MNPs@TA particles. To optimize the conditions for immobilization of arylsulfatase, the factors including the amount of arylsulfatase, immobilization time, and the buffer pH were examined for their effects on immobilization efficiency. The immobilization reactions were performed in a 2 ml system containing 10 mg of MNPs@TA beads, purified arylsulfatase, and buffer solution. To optimize the input of arylsulfatase, 10–60 μL (0.3–1.8 U) of arylsulfatase was used to immobilize on MNPs@TA in 50 mM sodium phosphate buffer (pH 7.0) with incubation at 5℃ for 3 hr. To optimize the immobilization time, 30 μL (0.9 U) of arylsulfatase was used to immobilize on MNPs@TA in 50 mM sodium phosphate buffer (pH 7.0) with incubation at 5℃ for 1–5 hr. To optimize the pH condition, 30 μL of arylsulfatase was used to immobilize on MNPs@TA in buffers with varying pH with incubation at 5℃ for 3 hr. The buffers examined included 50 mM sodium acetate‐acetic acid (pH 4.0–6.0), 50 mM sodium phosphate (pH 6.0–7.0), 50 mM Tris‐HCl (pH 7.0–9.0), and 50 mM glycine‐NaOH (pH 9.0–10.0). The recovery rate of enzyme immobilization was calculated based on the ratio of the enzyme activity of the immobilized arylsulfatase to that of the free arylsulfatase input. After optimization of the immobilization conditions, the immobilization of arylsulfatase was carried out by incubation of 0.9 U of arylsulfatase with 10 mg of MNPs@TA particles resuspended in 50 mM sodium phosphate buffer (pH 6.0) with a total volume of 2 ml at 5℃ for 3 hr. The MNPs@TA particles with immobilized arylsulfatase (MNPs@TA@ARS) were washed three time with deionized water. The beads were collected using permanent magnet and stored at 4℃ after freeze drying.

### 2.4 Arylsulfatase activity assay

The enzymatic activities of the free and immobilized arylsulfatases were determined using the chromogenic substrate *p*NPS following the method described previously (Zhu et al., 2018). Specifically, the substrate *p*NPS was prepared in 50 mM Tris‐HCl buffer with pH 8.0 and pH 7.5 for the free and immobilized arylsulfatase activity assays, respectively. 20 μl of purified arylsulfatase or 20 μl of Tris‐HCl buffer (50 mM, pH 7.5) containing 10 mg of the immobilized arylsulfatase was added to 80 μl of 20 mM *p*NPS prepared as mentioned above. The absorbance at 410 nm was recorded and converted to the units of enzyme activity defined as the amount of enzyme required to produce 1 μmol of *p*‐nitrophenyl per minute under the assay conditions.

### 2.5 *SEM*, TEM, Nitrogen adsorption, and FTIR assays

The particle size and surface property of MNPs@TA before and after immobilization of arylsulfatase were examined by *SEM* (Sigma 300, Carl Zeiss, Germany) and TEM (JEM‐2100F, JEOL Ltd., Japan). The nitrogen adsorption of MNPs@TA@ARS and MNPs@TA was determined by multi station extended specific surface and porosity analyzer ASAP2460 (Mcmurretik (Shanghai) Instrument Co., Ltd., China). The details of molecular structure of the MNPs@TA, MNPs@TA@ARS and the free arylsulfatase were characterized by FTIR (Nicolet Magna‐IR 170, Japan). The assays above were carried out following standard protocols and the instrument operational instructions.

### 2.6 Enzymatic parameters and tolerance to suboptimal conditions

To inquire into the optimal temperature and pH of the immobilized arylsulfatase, the effects of temperature and pH on the activity of the immobilized arylsulfatase against *p*NPS were examined. The enzymatic activities were monitored for the immobilized arylsulfatase in response to temperatures ranging from 30℃ to 65℃ and pH varying from 4.0 to 10.0 following the arylsulfatase activity assay described above. The activity of the immobilized arylsulfatase under certain condition was normalized to its maximum activity referring to the activity under optimal temperature or optimal pH. The kinetic parameters of *K*_m_ and *V*_max_ of the immobilized arylsulfatase were obtained by double‐reciprocal plotting of the enzymatic activities over varying concentrations of *p*NPS (0.1–3.0 mM) under optimal conditions. The thermal stabilities of the free and immobilized arylsulfatases were evaluated by measuring the residual activities of the enzymes exposed to 15–55℃ for 1 hr. The pH stabilities of these enzymes were monitored against the exposure to pH 4.0–10.0 at 4℃ for 1 hr. The residual activities were normalized to the initial activity which was defined as 100%.

### 2.7 Storage stability and reusability

The storage stabilities of the free and immobilized arylsulfatases were characterized by monitoring the residual enzyme activity every 5 days during the storage at 4℃ up to 25 days. The residual enzyme activities were normalized to the initial activity. The reusability of the immobilized arylsulfatase was evaluated by measuring the enzymatic activity over 6 successive applications. The beads were recovered by applying magnetic field and washed five times with 50 mM Tris‐HCl (pH 7.5) after each use.

### 2.8 Effects of various additives on enzyme activity

The effects of some additives on the arylsulfatase activity were examined. The metal ions including Na^+^, K^+^, Ca^2+^, Mg^2+^, Zn^2+^, Cu^2+^, Fe^2+^, Cd^2+^, Al^3+^, and Fe^3+^ were tested at the final concentrations of 1 mM or 10 mM, respectively. The detected inhibitors were ethylenediaminetetraacetic acid (EDTA), β‐mercaptoethanol (β‐ME), dithiothreitol (DTT), and phenylmethylsulfonyl fluoride (PMSF) at the final concentrations of 1 mM or 10 mM, respectively. The assayed detergents included sodium dodecyl sulfate (SDS), Tween‐20, Tween‐80, and Triton X‐100 at the final concentrations of 0.1% (w/v or v/v) or 1% (w/v or v/v), respectively. The effects of arylsulfatase to these additives were analyzed after incubation with each component at 4℃ for 30 min by recording the residual enzyme activity relative to the initial activity.

### 2.9 Agar desulfation and its physicochemical properties

The immobilized arylsulfatase was applied to remove the sulfate groups in agar. 4 g of agar (Guangdong Huankai Microbial Sci. & Tech. Co., Ltd., China) was suspended in 100 ml of 50 mM Tris‐HCl buffer (pH 7.5) and incubated with the immobilized or free arylsulfatase (80 U) at 50℃ for 6 hr with agitation at 100 rpm. After the reaction, the immobilized enzyme was collected using permanent magnet, and the free enzyme was inactivated at 90℃ for 5 min. The samples of agar pellets were collected by centrifugation and washed with ultrapure water for five times. After freeze drying, the sulfate content in the immobilized or free arylsulfatase‐treated agar was determined by ion chromatography (ICS‐2100; Dionex, USA). The untreated agar and commercial agarose (Hydragene Co. Ltd., USA) were included as controls to evaluate the performance of the immobilized arylsulfatase treatment. The gel strength, 3,6‐AG content, the melting, dissolving, and gelling temperatures, viscosity, transparency, whiteness, and ash were determined with reference to the methods described by Xiao et al., 2019.

### 2.10 Agar quality and DNA gel electrophoresis

The agar powder was coated with gold powder under vacuum condition and spread on conductive tape for examination under *SEM*. Digital images of the immobilized arylsulfatase‐treated agar, the untreated agar, and the commercial agarose were captured to show their surface morphology and physical property. For gel electrophoresis, 1% (w/v) of agar or agarose gel was prepared by dissolving the agar or agarose powder in 0.5 × TAE (Tris‐acetate‐EDTA) buffer. DNA ladders (New England BioLabs, USA) were separated on gels at 5 volts/cm for 60 min. The gels were stained with ethidium bromide for DNA shift visualization.

## 3 RESULTS AND DISCUSSION

### 3.1 Preparation of immobilized arylsulfatase

**FIGURE 1:**
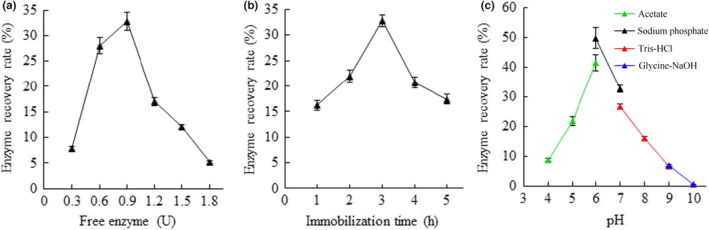
Optimization of the arylsulfatase immobilization conditions. (a) The input of free arylsulfatase. (b) The immobilization time. (c) The buffer pH. Sodium acetate (pH 4.0–6.0), sodium phosphate (pH 6.0–7.0), Tris‐HCl (pH 7.0–9.0), and glycine‐NaOH (pH 9.0–10.0) were examined. Values are mean ± *SD* of three biological replicates

To produce immobilized arylsulfatase, the magnetic Fe_3_O_4_ nanoparticles (MNPs) were synthesized and functionalized with tannic acid, and the mutant arylsulfatase K253H/H260L was purified from the heterologous expression in *E. coli*. The attached hydroxyl groups conferred by tannic acid molecules on the nanoparticle surface body provide the functional groups for enzyme immobilization through hydrogen bonding, electrostatic attraction, and potential covalent bonding (Abouelmagd et al., 2016), in addition to the improvement of MNPs hydrophilicity and dispensability (Qi et al., 2019). To achieve the maximal recovery rate of the free arylsulfatase through immobilization onto the tannic acid functionalized MNPs (MNPs@TA), the immobilization system was optimized against the amount of arylsulfatase, the immobilization time, and the buffer pH. The results showed that the optimal immobilization could be obtained by immobilization of 0.9 U of arylsulfatase on 10 mg of MNPs@TA particles (Figure 1a) for 3 hr (Figure 1b) in 50 mM sodium phosphate buffer (pH 6.0) (Figure 1c), leading to about 50% recruitment of the free arylsulfatase with the enzyme activity of the immobilized arylsulfatase 44.9 U/g.

It is noteworthy that the immobilization efficiency varies significantly under different pH conditions, with markedly reduced recovery rate at pH 5.0 and pH 7.0. The pH affects the ionization and electrical charge status of the molecules in the immobilization reaction system and the chemical stability of the enzyme, thus playing determinative role in the manner of interactions between the arylsulfatase and tannic acid in addition to the enzyme availability. The polyphenolic tannic acid is acidic and presumably negatively charged at pH 6.0. The pI (isoelectric point) value of the recombinant arylsulfatase is predicted to be 6.57 by using the compute pI/Mw tool (web.expasy.org), as such the arylsulfatase is presumably positively charged at pH 6.0. A similar application was documented for immobilization of trypsin on tannic acid functionalized Fe_3_O_4_ nanoparticles under pH 7.5 (Atacan & Ozacar, 2015). The exact ionization status of the arylsulfatase would be determined by the exposure of the side groups of amino acids to the aqueous buffer solution. In the meantime, the hydroxyl groups of tannic acid have great potentials for hydrogen bonding formation with functional groups in the arylsulfatase such as amide and amine groups. In addition, the carboxyl group of tannic acid could contribute to the covalent bonding of the enzyme molecules (Atacan & Ozacar, 2015). Nonetheless, the successful immobilization of arylsulfatase on MNPs@TA can attribute to the orchestrated effects of covalent and noncovalent interactions including electrostatic attraction and hydrogen bonding.

### 3.2 *SEM*, TEM, nitrogen adsorption, and FTIR characterization

**FIGURE 2:**
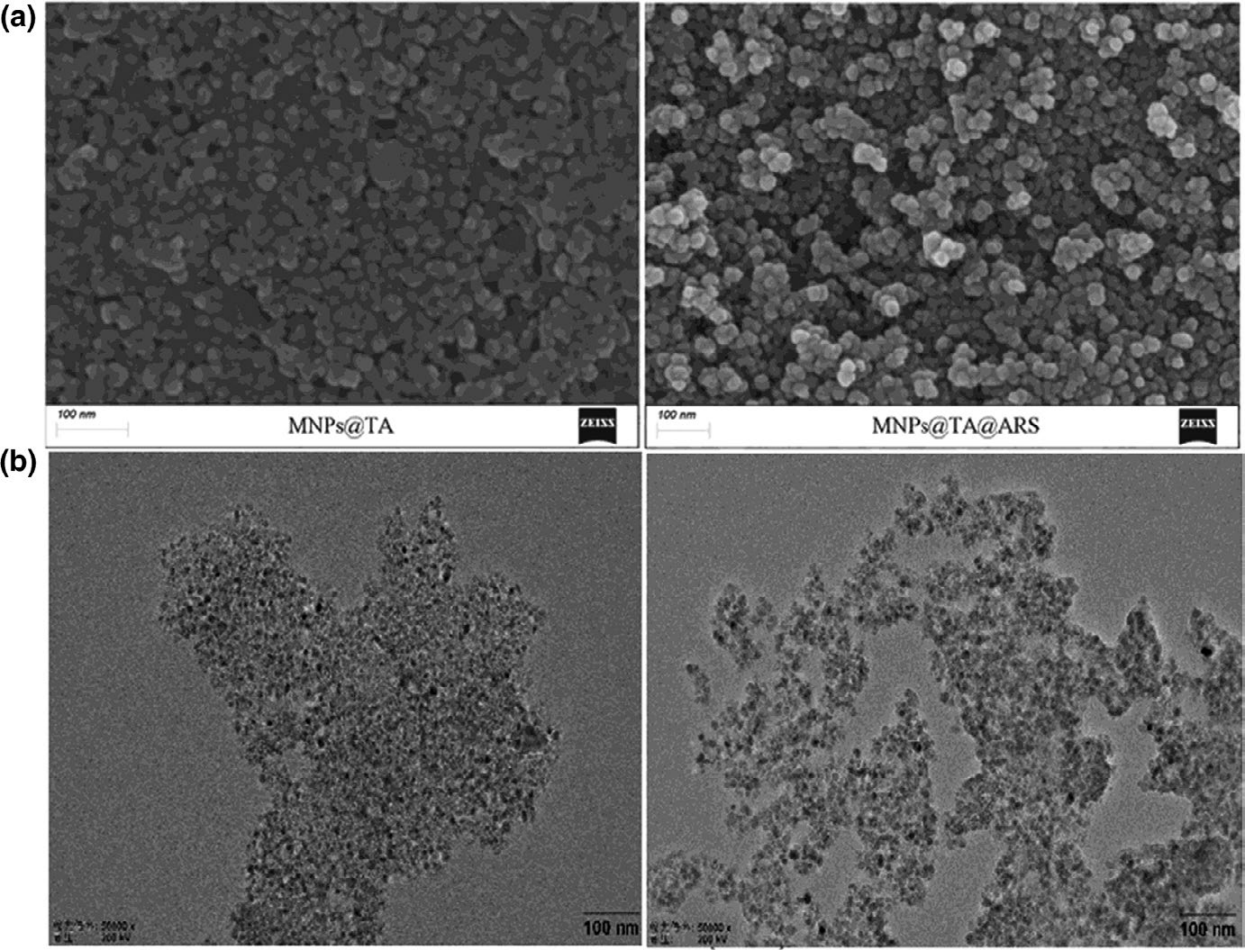
*SEM* and TEM analysis of the arylsulfatase immobilization. (a) *SEM* images of MNPs@TA particles (left) and MNPs@TA@ARS (right). (b) TEM images of MNPs@TA particles (left) and MNPs@TA@ARS (right)

**FIGURE 3:**
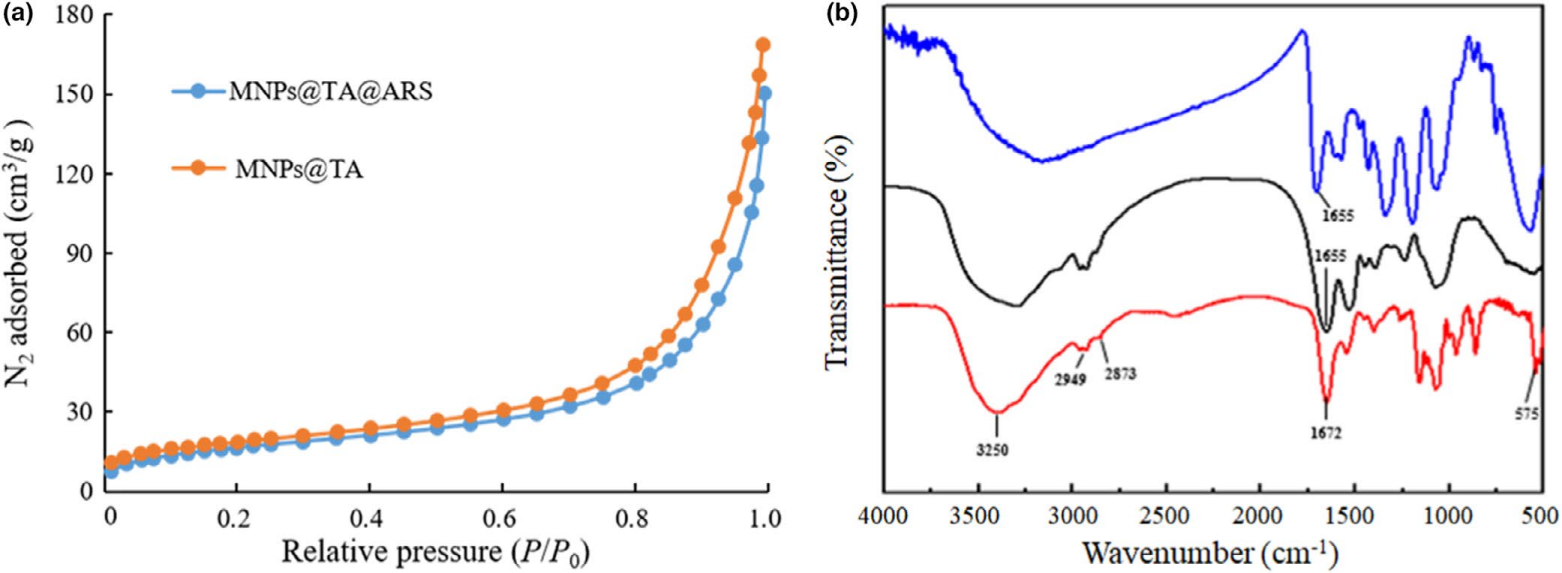
Nitrogen adsorption and FTIR analysis of the arylsulfatase immobilization. (a) Nitrogen adsorption curves of MNPs@TA (orange) and MNPs@TA@ARS (blue). (b) FTIR spectra of MNPs@TA (red), MNPs@TA@ARS (dark), and free arylsulfatase (blue)

To reveal the surface morphology and size distribution of the synthesized MNPs@TA before and after immobilization of arylsulfatase, *SEM* was performed and the images were recorded. The results showed that the dense dark approximately spherical particles of MNPs@TA (Figure 2a; left) and the relatively less compact pale particles with the immobilized arylsulfatase (Figure 2a; right), with an average diameter of about 15 nm, were observed, indicating the formation of ultra‐small nanoparticles after immobilization (Ansari et al., 2019). The results of TEM illustrated that compared to the carrier MNPs@TA (Figure 2b; left), the structure of the immobilized arylsulfatase (Figure 2b; right) was not so dense due to the combination of MNPs@TA and arylsulfatase. The nitrogen adsorption isotherms of MNPs@TA and MNP@TA@ARS were presented in Figure 3a. The quantity adsorbed of MNP@TA was slightly higher than that of MNP@TA@ARS at the relative pressures (*P*/*P*_0_) of 0.4–1.0. The sharp rise in the isotherms at high relative pressures (*P*/*P*_0_ near 1) indicated the existence of large mesopores and macropores in these samples (Sen et al., 2003). In addition, FTIR was performed to unveil the surface chemistry of MNPs@TA and the immobilization of arylsulfatase. The bands below 700 cm^‐1^ obtained in the spectra of MNP@TA (Figure 3b; red) and MNP@TA@ARS (Figure 3b; dark) represent the vibrations of Fe‐O bonds in Fe_3_O_4_ (Atacan & Ozacar, 2015). The absorption bands between 1,600 cm^−1^ and 1,400 cm^−1^ are ascribed to the aromatic ‐C = C‐ bonds (Atacan and Özacar, 2015). The broad peak at about 3,250 cm^‐1^ is the ‐OH stretching of tannic acid (Kim & Kim, 2003, Özacar et al., 2008). The presence of 2,949 cm^‐1^ and 2,873 cm^‐1^ peaks represent the stretching vibrations of ‐O‐H and ‐C‐H, respectively, from the conjugated tannic acid molecules (Atacan & Ozacar, 2015). The spectra patterns suggested the presence of Fe_3_O_4_ particles and the coating by tannic acid. The spectra of MNPs@TA@ARS (Figure 3b; dark) and the free arylsulfatase (Figure 3b; blue) appear the characteristic band at 1655 cm^‐1^, representing the C = O stretching vibration of amide I which is commonly found in the protein (Soares et al., 2020). These results substantiate the success of immobilization of the arylsulfatase on the surface of tannic acid functionalized Fe_3_O_4_ nanoparticles.

### 3.3 Enzymatic properties and stability

**FIGURE 4:**
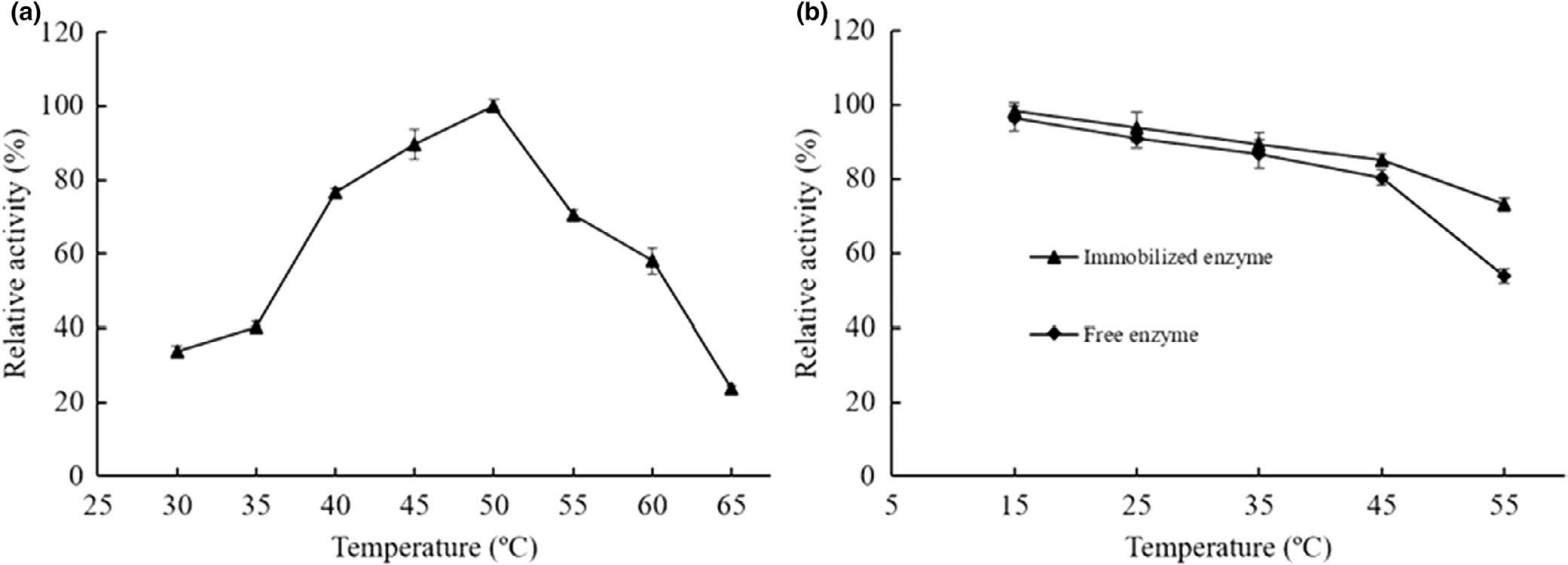
Effect of temperature on activity and stability of the immobilized arylsulfatase. (a) The temperature dependence. (b) The thermal stability. Values are mean ± *SD* of three biological replicates

**FIGURE 5:**
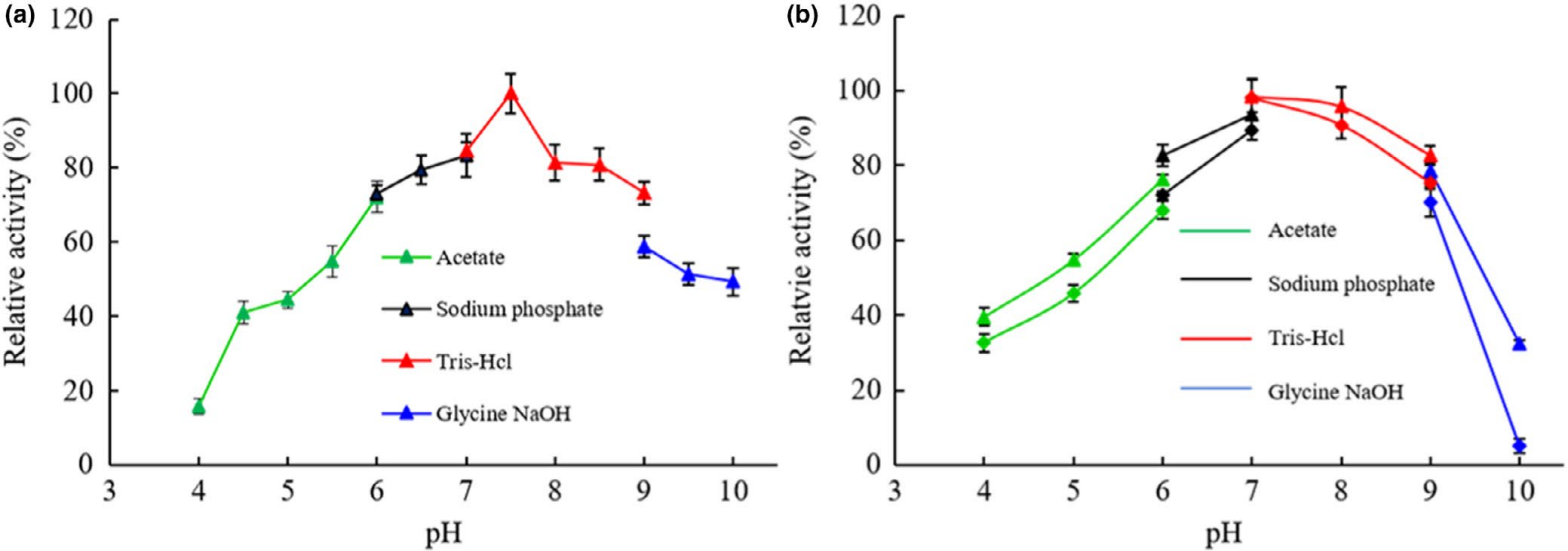
Effect of pH on activity and stability of the immobilized arylsulfatase. (a) The pH dependence. (b) The pH stability. Triangles represent the immobilized enzyme, and diamonds represent the free enzyme. Values are mean ± *SD* of three biological replicates

The effects of temperature and pH on the enzyme activity of arylsulfatase against *p*NPS substrate were investigated to gain an insight into the temperature and pH dependence of the immobilized enzyme. Based on the enzyme activities of the immobilized arylsulfatase under different temperature and pH conditions, the optimal temperature and pH were determined to be 50ºC (Figure 4a) and pH 7.5 (Figure 5a), respectively. Compared to the optimal temperature 55ºC and optimal pH 8.0 of the free arylsulfatase (Zhu et al., 2018), the immobilized arylsulfatase demonstrated shifted temperature and pH dependence, which implies the molecular interactions between the arylsulfatase and the phenolic hydroxyl groups or the free hydroxyl groups may affect the enzyme conformational structure and the microenvironment pH condition. It seemed contradictory to the general observation of acidic shift of immobilization of an enzyme on positively charged support (Abdel‐Naby et al., 1999), while MNPs@TA at pH 7.5 is presumably negatively charged, suggesting the interactions play more roles in shaping the enzyme conformation and affecting the pH dependency of the immobilized enzyme. Compared to the free arylsulfatase, the immobilized arylsulfatase exhibited moderately enhanced thermal stability (Figure 4b) and pH stability (Figure 5b) with stronger retained enzyme activity after exposure to the different temperatures and pH. After exposure at 55℃ for 1 hr, the immobilized arylsulfatase retained 73.0% of its original activity, while the free enzyme maintained the residual activity of 53.8% (Figure 4b). The residual activities of the immobilized and free arylsulfatases were 54.7% and 45.7% after exposure at pH 5.0 for 1 hr, respectively (Figure 5b). After the treatment at pH 10.0, the immobilized arylsulfatase maintained the residual activity of 32.4%, while the free enzyme was almost inactivated (Figure 5b). A plausible explanation is that the immobilization of the arylsulfatase on the support confers the relatively rigid conformation against the thermal disturbance and enhances the buffer capacity against the exposure to suboptimal pH leading to the denaturation of the enzyme (Guzik et al., 2014).

**FIGURE 6:**
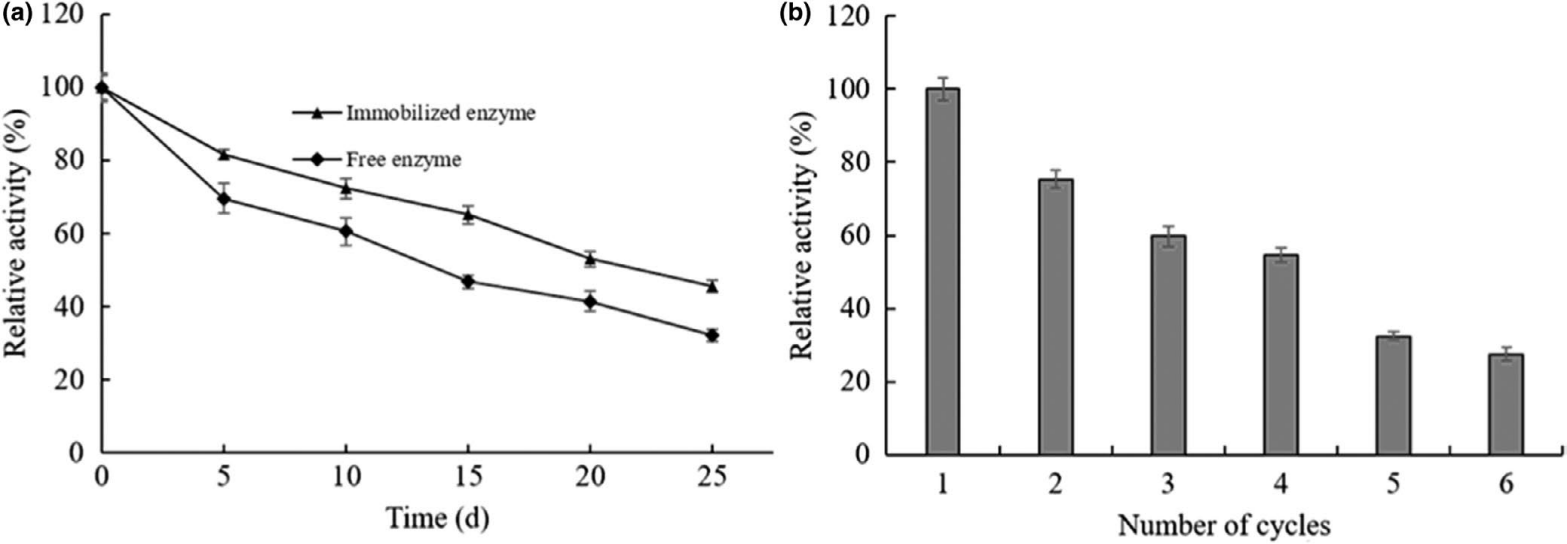
Storage stability and reusability of the immobilized arylsulfatase. (a) The storage stability. (b) The reusability. Values are mean ± *SD* of three biological replicates

The results of the storage stability and reusability for the immobilized arylsulfatase showed that the immobilized arylsulfatase possessed extended storage stability at 4℃ over the free arylsulfatase (Figure 6a), and demonstrated modest reusability with the residual activity up to 28.8% of its initial activity after 6 times of uses (Figure 6b).

**FIGURE 7:**
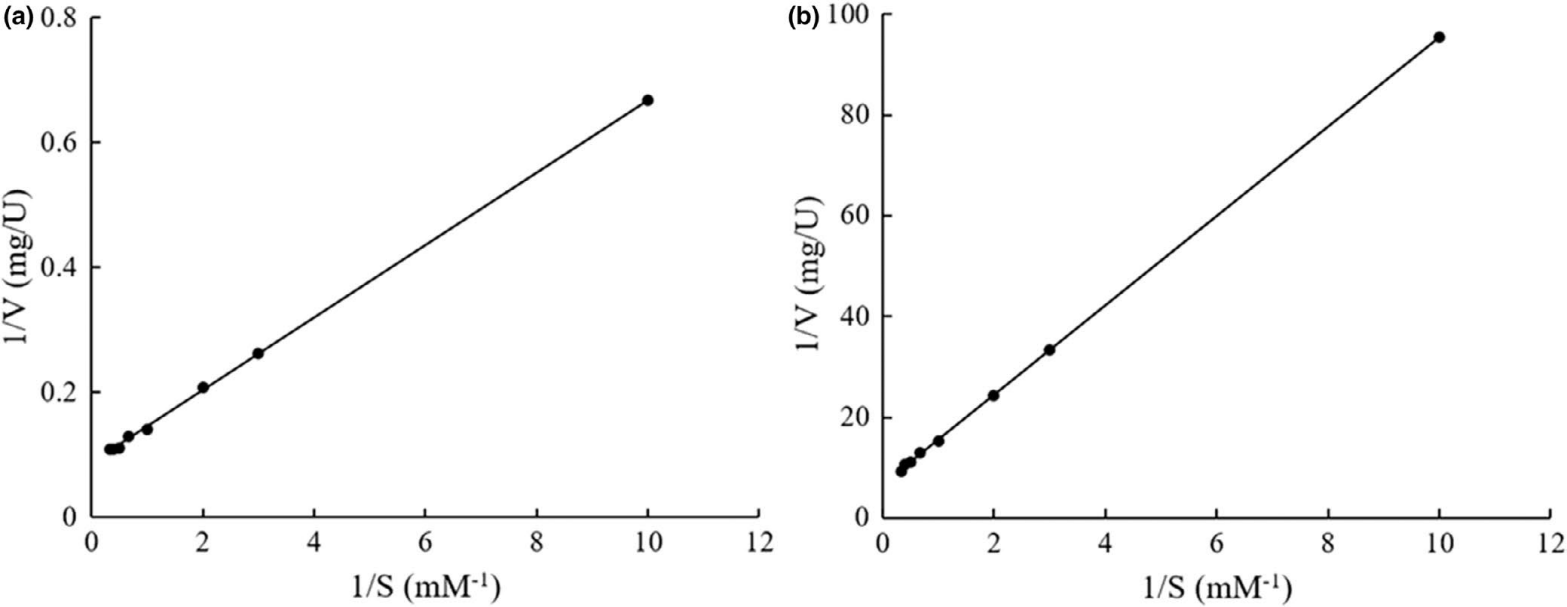
The Lineweaver‐Burk plots of the free arylsulfatase (a) and the immobilized arylsulfatase (b)

Under the optimal assay conditions, the enzyme activities of the immobilized arylsulfatase and the free enzyme were 0.14 U/mg and 10.91 U/mg, respectively. The Lineweaver‐Burk plots of the free and immobilized arylsulfatases were shown in Figure 7, and their comparisons of the enzymatic kinetic parameters were shown in Table 1. The catalytic parameters *K*_m_ and *V*_max_ of the immobilized arylsulfatase against the substrate *p*NPS under the optimal temperature and pH were 1.33 mM and 0.15 μmol/(mg·min), respectively, which were evidently deviated from the *K*_m_ (0.66 mM) and *V*_max_ (11.37 μmol/(mg·min)) of the free arylsulfatase (Table 1). It is not surprising to detect the altered apparent *K*_m_ and *V*_max_ after enzyme immobilization due to the heterogeneous microenvironment hatching the enzymatic reaction (Robinson, 2015). The apparent *K_m_* value of the immobilized arylsulfatase is higher than that of the free enzyme, indicating that the immobilized enzyme has a lower binding affinity for the substrate than the free one. This may be caused by the steric hindrance of the active site by the support, the loss of enzyme flexibility necessary for substrate binding, or diffusional resistance to solute transport near the particles of the support (Şahin et al., 2005). The apparent *V_max_* of the immobilized arylsulfatase is significantly lower than that of the free enzyme. This decrease in *V*_max_ might be attributed to limited accessibility of substrate molecules to the active sites of the enzyme and the interaction of the enzymes with the functional groups on the surface of beads or large areas of contact between enzyme and support (Keerti et al., 2014). Similar observations about the changes of apparent *K_m_* and *V_max_* values of immobilized enzyme have been reported in some other studies (Hassan et al., 2019; Raghu & Pennathur, 2018).

**TABLE 1:** Kinetic parameters of immobilized and free arylsulfatases

Enzyme	*K*_m_ (mM)	*V*_max_ (U/mg)
Immobilized arylsulfatase	1.33	0.15
Free arylsulfatase ^a^	0.66	11.37

^a^ The kinetic parameters of free arylsulfatase were from Zhu et al., 2018.

### 3.4 Resistance to the influences of additives

**FIGURE 8:**
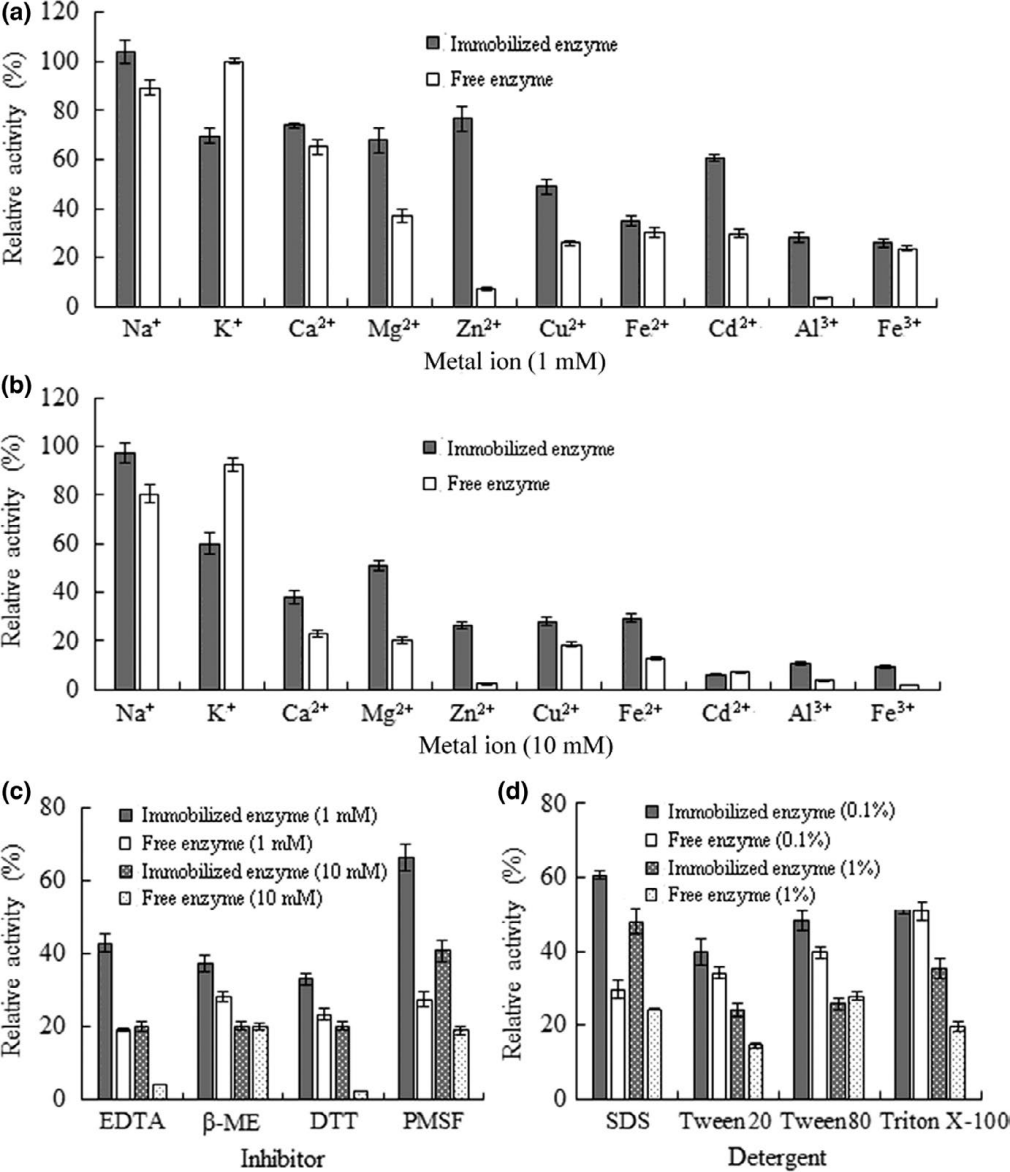
Effects of the additives on the activity of the immobilized arylsulfatase. (a) The metal ions at 1 mM. (b) The metal ions at 10 mM. (c) The inhibitors. (d) The detergents. Values are mean ± *SD* of three biological replicates

Ionic identity and strength play significant roles in determining enzyme activities through affecting the configuration of the active site and enzyme stability. To testify the resistance of the immobilized arylsulfatase to ionic challenges, the residual activities of the immobilized arylsulfatase were measured after incubation with various metal ions. The results showed that the immobilized arylsulfatase demonstrated enhanced tolerance to the inhibitory effects of metal ions except for K^+^ with concentration as high as 1 mM and 10 mM, as compared to the free arylsulfatase (Figure 8a and b). These may be due to the limited mobility of the protein chain after its immobilization (Xiao et al., 2017).

Moreover, similar trend was observed on the immobilized arylsulfatase with enhanced tolerance to these additives monitored (Figure 8c and d). Especially, the inhibitors of β‐ME, DTT, and EDTA displayed pronounced inhibitory effects on the activity of arylsulfatase (Figure 8c). The lack of thiol group or cysteine residue in the arylsulfatase implies that the actions of β‐ME and DTT are independent of thiol‐disulfide exchange but related to their effects on the conformation of active site directly or allosterically. The inhibitory effect of DTT against a cysteineless pigpen mutant protein was described in a previous study (Alliegro, 2000). The inhibitory effect of EDTA suggested that covalent ion could be involved in the maintenance of the arylsulfatase activity. Additionally, the immobilized arylsulfatase exhibited enhanced tolerance to the inhibitory effects of ionic detergent (SDS) and nonionic detergents (Tween 20, Tween 80, and Triton X‐100) over the free arylsulfatase (Figure 8d). Taken together, the immobilization of arylsulfatase on the solid matrix of magnetic nanoparticles imparts its conformational stability and better tolerance to the enzyme inhibitory factors.

### 3.5 Agar desulfation activity and gel quality

To explore the advantages of the immobilized arylsulfatase for practical application, the efficiency of removing sulfate groups from agar was examined. For the immobilized and free arylsulfatases, the desulfation rates of agar were 57.41% and 67.23%, respectively. The former had about 10% decline of the desulfation rate lower than the latter. Nonetheless, the enhanced storage stability and reusability, easy enzyme recovery will make the immobilized arylsulfatase a valuable candidate for the industrial applications. After treatment by the immobilized arylsulfatase, the sulfate content in the agar was significantly reduced reaching the level of about only 0.08% higher compared to its content in the agarose (Table 2). As a consequence, the 3,6‐AG content was increased to a level of 5.65% lower than that in the agarose. Other physical and visual characterizations indicated that the agar treated by the immobilized arylsulfatase had intermediate properties between the untreated agar and the commercial agarose (Table 2). The viscosity of the immobilized arylsulfatase‐treated agar was reduced by 5.54%, attributing to the decreased content of agaropectin. These results were consistent with the observation of increased 3,6‐AG content (Table 2). The optical clarity is an important property of agar products, providing better resolution to observations (Jaeger et al., 2015). The treatment by the immobilized arylsulfatase led to the increase in transparency by 3.40% and whiteness by 12.57% (Table 2), making the treated agar more applicable for gel electrophoresis and microbiological assays. Notably, the gel strength of the immobilized arylsulfatase‐treated agar was comparable to that of agarose, partly owing to the significantly reduced ash and sulfate contents, rendering the application of the immobilized arylsulfatase a promising prospective.

**TABLE 2:** The physical and chemical properties of the agar and agarose

Properties	Immobilized enzyme‐treated agar	Agar	Agarose
Sulfate content (%)	0.23 ± 0.01	0.54 ± 0.02	0.15 ± 0.01
Gel strength (g/cm^2^)	1,139.02 ± 35.56	833.37 ± 42.85	1,204.35 ± 52.82
3,6‐AG content (%)	33.23 ± 0.47	24.60 ± 1.04	38.88 ± 0.25
Melting temperature (°C)	93.30 ± 0.36	93.93 ± 0.12	94.00 ± 0.20
Dissolving temperature (°C)	95.93 ± 0.21	95.10 ± 0.14	97.45 ± 0.49
Gelling temperature (°C)	36.57 ± 0.49	36.40 ± 0.53	37.77 ± 0.38
Viscosity (cp)	32.53 ± 0.65	38.07 ± 0.12	28.83 ± 1.23
Transparency (%)	57.53 ± 0.50	54.13 ± 0.49	63.13 ± 0.78
Whiteness (%)	67.12 ± 1.44	54.55 ± 0.12	85.27 ± 0.16
Ash (%)	1.29 ± 0.10	2.97 ± 0.15	0.50 ± 0.02

**FIGURE 9:**
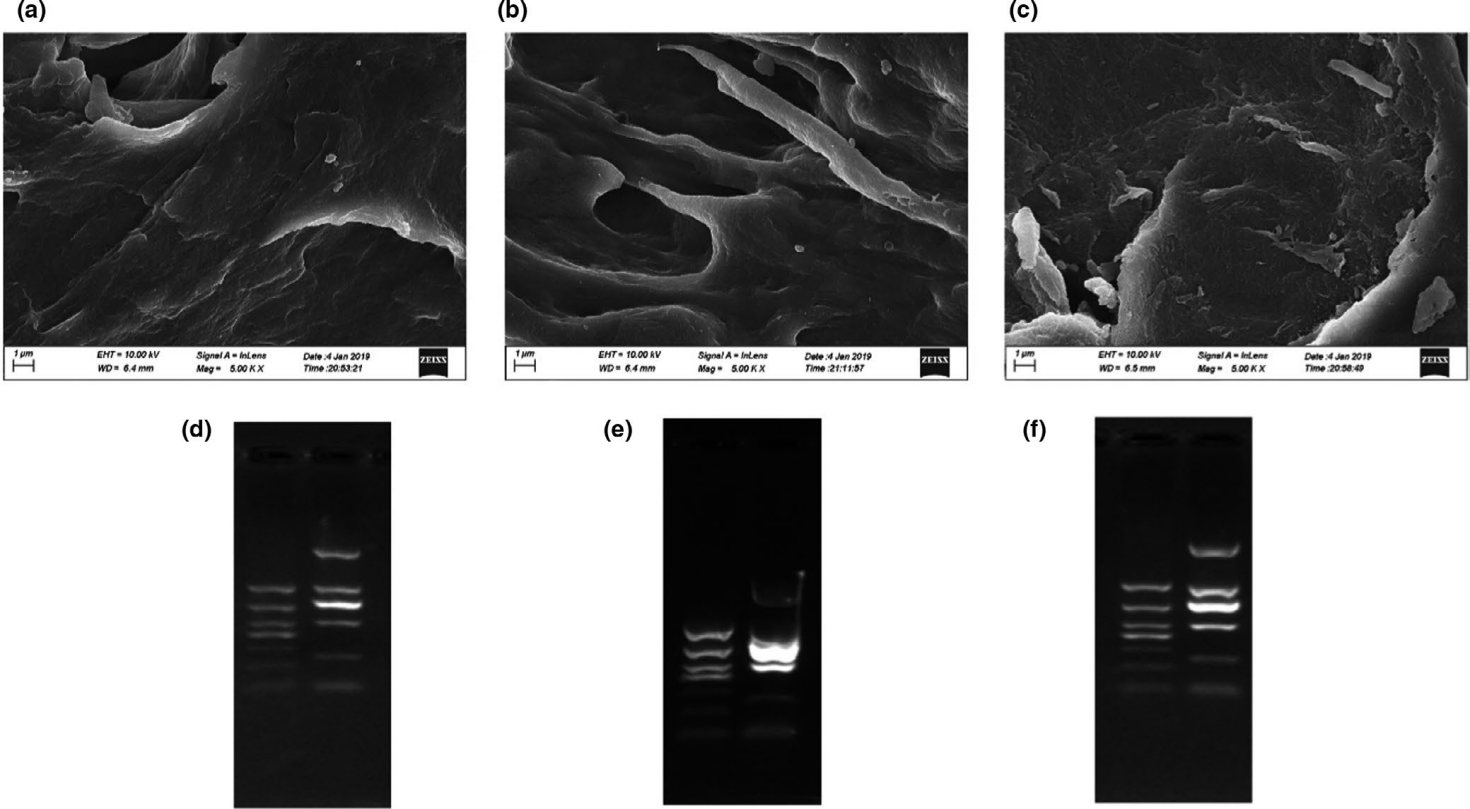
The agar quality examination by *SEM* and gel electrophoresis. The *SEM* images of the agar treated with the immobilized arylsulfatase (a), the untreated agar (b), and the agarose (c). The DNA electrophoresis on gels composed of the agar treated with the immobilized arylsulfatase (d), the untreated agar (e), and the agarose (f)

**FIGURE 10:**
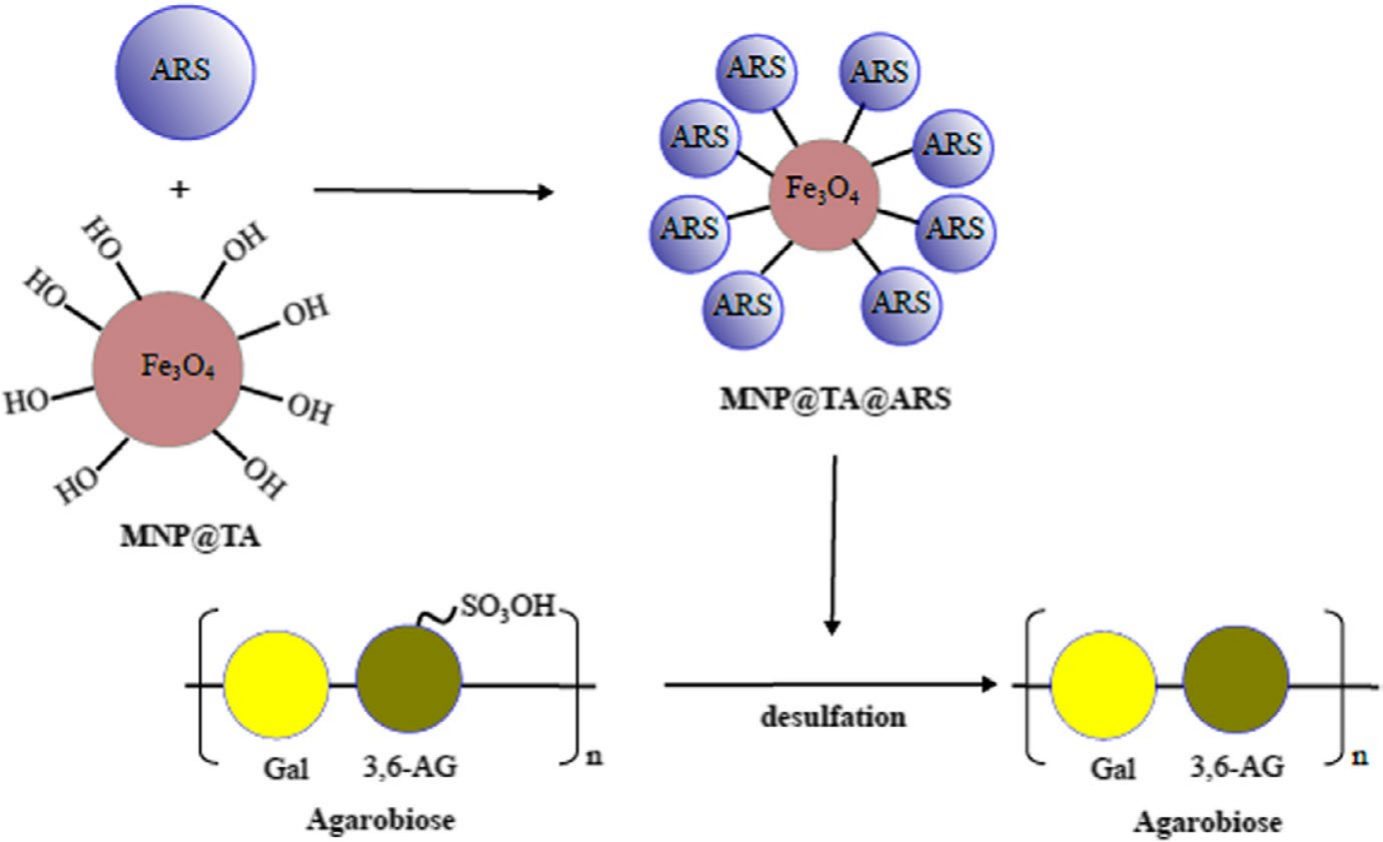
Schematic overview of the action of the immobilized arylsulfatase on agar

To further evaluate the desulfation performance of the immobilized arylsulfatase against agar, the resulting agar powder was examined under *SEM* for its physical appearance. The results showed that the immobilized arylsulfatase‐treated agar powder took on smooth surface and better adherence (Figure 9a), which was similar to the appearance of the agarose (Figure 9c). However, the agar powder without the arylsulfatase treatment displayed a rough surface (Figure 9b). The difference in morphology of the agar before and after the immobilized arylsulfatase treatment indicated the improved crosslinking contributing to the enhanced mechanical property (Shukla et al., 2011). Additionally, the agar quality was examined for its application in gel electrophoresis. The comparison between the DNA shift patterns indicated the ladders presented similar banding on the immobilized arylsulfatase‐treated agar (Figure 9d) and the commercial agar (Figure 9f), with better separation and higher resolution compared with the mobility pattern on the untreated agar (Figure 9e). Taken together, our findings suggest that it is a practical strategy to develop the immobilized arylsulfatase for its utilization in agar desulfation to improve the agar quality (Figure 10).

## 4 CONCLUSIONS

To sum up, the arylsulfatase was immobilized on tannic acid‐functionalized Fe_3_O_4_ nanoparticles and was utilized to perform desulfation against agar. *SEM* and FTIR assays supported the success of the arylsulfatase immobilization. The immobilization of the arylsulfatase imparted its stability, modest reusability, and better tolerance to the diverse additives that affect enzymatic activity. The immobilized arylsulfatase‐treated agar with significantly decreased sulfate content gave the gel quality comparable to that of commercial agarose. Our study will help further optimize and develop the immobilized arylsulfatase for its industrial application for agar and agarose production.

## CONFLICT OF INTEREST

The authors have no conflict of interest to disclose.

## ETHICAL APPROVAL

Ethics approval was not required for this research.

## Data Availability

Research data are not shared.

## References

[fsn32446-bib-0001] Abdel‐Naby, M. A., Sherif, A. A., El‐Tanash, A. B., & Mankarios, A. T. (1999). Immobilization of Aspergillus oryzae tannase and properties of the immobilized enzyme. Journal of Applied Microbiology, 87, 108–114.

[fsn32446-bib-0002] Abouelmagd, S. A., Meng, F., Kim, B. K., Hyun, H., & Yeo, Y. (2016). Tannic acid‐mediated surface functionalization of polymeric nanoparticles. ACS Biomaterials Science & Engineering, 2, 2294–2303. 28944286 10.1021/acsbiomaterials.6b00497 PMC5609506

[fsn32446-bib-0003] Abraham, A., Afewerki, B., Tsegay, B., Ghebremedhin, H., Teklehaimanot, B., & Reddy, K. S. (2018). Extraction of agar and alginate from marine seaweeds in Red Sea region. International Journal of Marine Biology and Research, 3, 1–8.

[fsn32446-bib-0004] Alliegro, M. C. (2000). Effects of dithiothreitol on protein ativity unrelated to thiol‐disulfide exchange: For consideration in the analysis of protein function with Cleland's reagent. Analytical Biochemistry, 282, 102–106. 10860505 10.1006/abio.2000.4557

[fsn32446-bib-0005] Ansari, S., Ficiarà, E., Ruffinatti, F. A., Stura, I., Argenziano, M., Abollino, O., Cavalli, R., Guiot, C., & D'Agata, F. (2019). Magnetic iron oxide nanoparticles: Synthesis, characterization and functionalization for biomedical applications in the central nervous system. Materials, 12, 465. 10.3390/ma12030465 PMC6384775 30717431

[fsn32446-bib-0006] Atacan, K., Çakiroğlu, B., & Özacar, M. (2017). Efficient protein digestion using immobilized trypsin onto tannin modified Fe3O4 magnetic nanoparticles. Colloids & Surfaces B: Biointerfaces, 156, 9–18. 28499203 10.1016/j.colsurfb.2017.04.055

[fsn32446-bib-0007] Atacan, K., & Özacar, M. (2015). Characterization and immobilization of trypsin on tannic acid modified Fe3O4 nanoparticles. Colloids & Surfaces B: Biointerfaces, 128, 227–236. 25686792 10.1016/j.colsurfb.2015.01.038

[fsn32446-bib-0008] Basso, A., & Serban, S. (2019). Industrial applications of immobilized enzymes—A review. Molecular Catalysis, 479, 110607.

[fsn32446-bib-0009] Bilal, M., Mahmood, S., Rasheed, T., & Iqbal, H. (2019). Bio‐catalysis and biomedical perspectives of magnetic nanoparticles as versatile carriers. Magnetochemistry, 5, 42.

[fsn32446-bib-0010] Cregut, M., & Rondags, E. (2013). New insights in agar biorefinery with arylsulphatase activities. Process Biochemistry, 48, 1861–1871.

[fsn32446-bib-0011] Fu, X. T., & Kim, S. M. (2010). Agarase: Review of major sources, categories, purification method, enzyme characteristics and applications. Marine Drugs, 8, 200–218. 20161978 10.3390/md8010200 PMC2817930

[fsn32446-bib-0012] Gardner, T. G., & Senwo, Z. N. (2019). Enzymatic hydrolysis of an organic sulfur compound. Advances in Enzyme Research, 7, 1–13.

[fsn32446-bib-0013] Guzik, U., Hupert‐Kocurek, K., & Wojcieszyńska, D. (2014). Immobilization as a strategy for improving enzyme properties‐application to oxidoreductases. Molecules, 19, 8995–9018. 24979403 10.3390/molecules19078995 PMC6271243

[fsn32446-bib-0014] Hassan, M., Yang, Q., & Xiao, Z. (2019). Covalent immobilization of glucoamylase enzyme onto chemically activated surface of κ‐carrageenan. Bulletin of the National Research Centre, 43, 102.

[fsn32446-bib-0015] Jaeger, P. A., McElfresh, C., Wong, L. R., & Ideker, T. (2015). Beyond agar: Gel substrates with improved optical clarity and drug efficiency and reduced autofluorescence for microbial growth experiments. Appllied and Environmental Microbiology, 81, 5639–5649. 10.1128/AEM.01327-15 PMC4510171 26070672

[fsn32446-bib-0016] Jiang, Z., Zhang, X., Wu, L., Li, H., Chen, Y., Li, L., Ni, H., Li, Q., & Zhu, Y. (2021). Exolytic products of alginate by the immobilized alginate lyase confer antioxidant and antiapoptotic bioactivities in human umbilical vein endothelial cells. Carbohydrate Polymers, 251, 116976. 33142553 10.1016/j.carbpol.2020.116976

[fsn32446-bib-0017] Keerti , Gupta, A., Kumar, V., Dubey, A. & Verma, A.K. (2014). Kinetic characterization and effect of immobilized thermostable β‐glucosidase in alginate gel beads on sugarcane juice. ISRN Biochemistry, 2014, 178498. 25969764 10.1155/2014/178498 PMC4392994

[fsn32446-bib-0018] Kim, S., & Kim, H. J. (2003). Curing behavior and viscoelastic properties of pine and wattle tannin‐based adhesives studied by dynamic mechanical thermal analysis and FT‐IR‐ATR spectroscopy. Journal of Adhesion Science and Technology, 17, 1369–1383.

[fsn32446-bib-0019] Mohamad, N. R., Marzuki, N. H., Buang, N. A., Huyop, F., & Wahab, R. A. (2015). An overview of technologies for immobilization of enzymes and surface analysis techniques for immobilized enzymes. Biotechnology, Biotechnological Equipment, 29, 205–220. 26019635 10.1080/13102818.2015.1008192 PMC4434042

[fsn32446-bib-0020] Özacar, M., Şengil, İ. A., & Türkmenler, H. (2008). Equilibrium and kinetic data, and adsorption mechanism for adsorption of lead onto valonia tannin resin. Chemical Engineering Journal, 143, 32–42.

[fsn32446-bib-0021] Qi, P., Luo, R., Pichler, T., Zeng, J., Wang, Y., Fan, Y., & Sui, K. (2019). Development of a magnetic core‐shell Fe3O4@TA@UiO‐66 microsphere for removal of arsenic(III) and antimony(III) from aqueous solution. Journal of Hazardous Materials, 378, 120721. 31200224 10.1016/j.jhazmat.2019.05.114

[fsn32446-bib-0022] Raghu, S., & Pennathur, G. (2018). Enhancing the stability of a carboxylesterase by entrapment in chitosan coated alginate beads. Turkish Journal of Biology, 42, 307–318. 30814894 10.3906/biy-1805-28 PMC6353290

[fsn32446-bib-0023] Robinson, P. K. (2015). Enzymes: Principles and biotechnological applications. Essays in Biochemistry, 59, 1–41. 26504249 10.1042/bse0590001 PMC4692135

[fsn32446-bib-0024] Şahin, F., Demirel, G., & Tümtürk, H. (2005). A novel matrix for the immobilization of acetylcholinesterase. International Journal of Biological Macromolecules, 37, 148–153. 16274740 10.1016/j.ijbiomac.2005.10.003

[fsn32446-bib-0025] Sen, T., Tiddy, G., Casci, J., & Anderson, M. (2003). Macro‐cellular silica foams: Synthesis during the natural creaming process of an oil‐in‐water emulsion. Chemical Communications, 17, 2182–2183. 10.1039/b303349j 13678191

[fsn32446-bib-0026] Shukla, M., Kumar, M., Prasad, K., Reddy, C. R. K., & Jha, B. (2011). Partial characterization of sulfohydrolase from Gracilaria dura and evaluation of its potential application in improvement of the agar quality. Carbohydrate Polymers, 85, 157–163.

[fsn32446-bib-0027] Sirisha, V. L., Jain, A., & Jain, A. (2016). Enzyme immobilization: An overview on methods, support material, and applications of immobilized enzymes. Advance in Food and Nutrition Research, 79, 179–211. 10.1016/bs.afnr.2016.07.004 27770861

[fsn32446-bib-0028] Soares, A. M. B. F., Gonçalves, L. M. O., Ferreira, R. D. S., de Souza, J. M. , Fangueiro, R., Alves, M. M. M., Carvalho, F. A. A., Mendes, A. N., & Cantanhêde, W. (2020). Immobilization of papain enzyme on a hybrid support containing zinc oxide nanoparticles and chitosan for clinical applications. Carbohydrate Polymers, 243, 116498. 32532402 10.1016/j.carbpol.2020.116498

[fsn32446-bib-0029] Stressler, T., Seitl, I., Kuhn, A., & Fischer, L. (2016). Detection, production, and application of microbial arylsulfatases. Appllied Microbiology and Biotechnology, 100, 9053–9067. 10.1007/s00253-016-7838-4 27654655

[fsn32446-bib-0030] Toennes, S. W., & Maurer, H. H. (1999). Efficient cleavage of conjugates of drugs or poisons by immobilized beta‐glucuronidase and arylsulfatase in columns. Clinical Chemistry, 45, 2173–2182. 10585350

[fsn32446-bib-0031] Torres, M. D., Flórez‐Fernández, N., & Domínguez, H. (2019). Integral utilization of red seaweed for bioactive production. Marine Drugs, 17, 314. 10.3390/md17060314 PMC6627364 31142051

[fsn32446-bib-0032] Wang, X., Duan, D., & Fu, X. (2016). Enzymatic desulfation of the red seaweeds agar by Marinomonas arylsulfatase. International Journal of Biological Macromolecules, 93, 600–608. 27521846 10.1016/j.ijbiomac.2016.08.031

[fsn32446-bib-0033] Xiao, Q., An, D., Zhang, C., Weng, H., Zhang, Y., Chen, F., & Xiao, A. (2019). Agar quality promotion prepared by desulfation with hydrogen peroxide. International Journal of Biological Macromolecules, 145, 492–499. 31883896 10.1016/j.ijbiomac.2019.12.206

[fsn32446-bib-0034] Xiao, Q., Yin, Q., Ni, H., Cai, H., Wu, C., & Xiao, A. (2017). Characterization and immobilization of arylsulfatase on modified magnetic nanoparticles for desulfation of agar. International Journal of Biological Macromolecules, 94, 576–584. 27746358 10.1016/j.ijbiomac.2016.10.029

[fsn32446-bib-0035] Zdarta, J., Meyer, A., Jesionowski, T., & Pinelo, M. (2018). A general overview of support materials for enzyme immobilization: Characteristics, properties, practical utility. Catalysts, 8, 92.

[fsn32446-bib-0036] Zhang, Y., Fu, X., Duan, D., Xu, J., & Gao, X. (2019). Preparation and characterization of agar, agarose, and agaropectin from the red alga Ahnfeltia plicata. Journal of Oceanology and Limnology, 37, 815–824.

[fsn32446-bib-0037] Zhu, Y., Qiao, C., Li, H., Li, L., Xiao, A., Ni, H., & Jiang, Z. (2018). Improvement thermostability of Pseudoalteromonas carrageenovora arylsulfatase by rational design. International Journal of Biological Macromolecules, 108, 953–959. 29113885 10.1016/j.ijbiomac.2017.11.014

